# Cost-effectiveness evaluation of risk-based breast cancer screening in Urban Hebei Province

**DOI:** 10.1038/s41598-023-29985-z

**Published:** 2023-02-27

**Authors:** Jin Shi, Yazhe Guan, Di Liang, Daojuan Li, Yutong He, Yunjiang Liu

**Affiliations:** 1 Cancer Institute, The Tumor Hospital of Hebei Province, The Fourth Hospital of Hebei Medical University, Shijiazhuang, 050011 Hebei People’s Republic of China; 2 Department of Breast Cancer Center, The Tumor Hospital of Hebei Province, The Fourth Hospital of Hebei Medical University, Shijiazhuang, 050011 Hebei People’s Republic of China

**Keywords:** Cancer, Oncology

## Abstract

To evaluate the implementations of Cancer Screening Program in Urban Hebei and to model the cost-effectiveness of a risk-based breast Cancer Screening Program. Women aged 40–74 years were invited to participate the Cancer Screening Program in Urban Hebei form 2016 to 2020 by completing questionnaires to collect information about breast cancer exposure. Clinical screening including ultrasound and mammography examination were performed. We developed a Markov model to estimate the lifetime costs and benefits, in terms of quality-adjusted life years (QALY), of a high-risk breast Cancer Screening Program. Nine screening strategies and no screening were included in the study. The age-specific incidence, transition probability data and lifetime treatment costs were derived and adopted from other researches. Average cost-effectiveness ratios (ACERs) were estimated as the ratios of the additional costs of the screening strategies to the QLYG compared to no screening. Incremental cost-effectiveness ratios (ICERs) were calculated based on the comparison of a lower cost strategies to the next more expensive and effective strategies after excluding dominated strategies and extendedly dominated strategies. ICERs were used to compare with a willingness-to-pay (WTP) threshold. Sensitivity analysis was explored the influence factors. A total of 84,029 women completed a risk assessment questionnaire, from which 20,655 high-risk breast cancer females were evaluated, with a high-risk rate of 24.58%. There were 13,392 high-risk females completed the screening program, with participation rate was 64.84%. Undergoing ultrasound, mammography and combined screening, the suspicious positive detection rates were 15.00%, 9.20% and 19.30%, and the positive detection rates were 2.11%, 2.76% and 3.83%, respectively. According to the results by Markov model, at the end of 45 cycle, the early diagnosis rates were 55.53%, 60.68% and 62.47% underwent the annual screening by ultrasound, mammography and combined, the proportion of advanced cancer were 17.20%, 15.85% and 15.36%, respectively. Different screening method and interval yield varied. In the exploration of various scenarios, annual ultrasound screening is the most cost-effective strategy with the ICER of ¥116,176.15/QALY. Sensitivity analyses demonstrated that the results are robust. Although it was not cost effective, combined ultrasound and mammography screening was an effective strategy for higher positive detection rate of breast cancer. High-risk population-based breast cancer screening by ultrasound annually was the most cost-effective strategy in Urban Hebei Province.

## Introduction

According to the GLOBOCAN 2020, breast cancer is the most commonly diagnosed cancer in females, with age standardized incidence and mortality rates of 47.8 × 10^5^ and 13.6 × 10^5^, respectively^1,2^. Female breast cancer is now the most common cancer in China, the incidence is increasing twice as fast as the rate of global, it will continue to increase and there are no signs that this trend will stop by 2030^3^. The diagnosed mean age for breast cancer is 45–55 years old in Chinese women, and it is younger almost 10 years than the women in western developed regions. At an early stage diagnosed, breast cancer patients have a better prognosis, but at an advanced stage diagnosed, patients have a worse prognosis^4–6^. Detecting and diagnosing at an early stage by breast cancer screening strategy has been confirmed to ease disease burden and improve survival outcomes^7–11^.

To explore screening program for breast cancer, majority countries published the screening and guidelines and standards. In the USA, all guidelines agreed that the females with average risk should perform routine mammography screening, despite differences from departments^12^. In European, the guidelines recommend organized mammography screening programs for 40–75 years old women with at an average risk^13^. Several large trials of breast cancer screening, including the population-based Cancer Screening Program in Urban China (CanSPUC), had been established and implemented in China^14^. The preliminary results indicated that screening detected significantly higher proportions of early-stage breast cancer and tumor sizes < 2 cm among both urban and rural than in the clinic, which indicated that more effective therapy could be selected to improve prognosis^15^. Hebei was one of the first nine provinces participated in the CanSPUC, which targeted five types of cancer screening that are most prevalent in urban regions, including female breast cancer. Eligible participants were recruited and invited to participate the cancer screening willingly free of charge. For female breast screening, the individuals evaluated as breast cancer high risk by Clinical Cancer Risk Score System were recommended to undergo subsequent ultrasound combined with mammography detection in the designated tertiary-level hospitals^16^.

Breast cancer screening is regard as the effective method to enhance the early diagnosis rates^17,18^. As is well known, on the one hand, the breast cancer mammography screening strategy by population-based had been widely implemented in developed regions for few decades. A systematic review reported eighteen researches reported that breast cancer screening by mammography was regarded as a cost-effective strategy and almost 70% researches were implemented in upper income and middle income regions^19^. On the other hand, perhaps because of less developed regions have limited resources compared to developed regions, whether the breast cancer screening by mammography could be effective and cost-effectiveness is still unclear, and even an intractable problem^20,21^. Researches in some developing and less developed regions including China have revealed that breast cancer screening strategy by mammography is not attractively in terms of economic^22–26^. Considering Chinese women tend to have smaller and more dense breasts, the sensitivity of mammography correlates negatively with breast density and is especially limited in younger, but ultrasound screening was considered to have the potential to detect the small nodules^27–29^. Therefore, what’s the screening strategies and interval are more effective and cost-effective in Chinese females, especially in Hebei Province requires further health and economics evaluation.

In this current study, the purpose is to introduce Hebei’s assessment system for high risk-based screening strategy and description the baseline information. Meanwhile, we explored the health economic evaluation in different strategies and interval of breast cancer screening strategy by Markov model.

## Materials and methods

### Data source and screening process of high-risk breast cancer population

In order to evaluate the risk of breast cancer for individuals, health professionals invited women aged 40–74 years to the health facilities and hospitals, then used paper-based questionnaires to collect information about breast cancer risk exposure. The “Harvard Cancer Index online tool” was used by the health professionals to process the collected information. The tool calculates individual cancer scores by giving risk scores to exposures, which including family history, height, age of first period, age of first birth, number of births, age at menopause, use of oral contraceptives, estrogen replacement and so on^30,31^.

**Figure 1:**
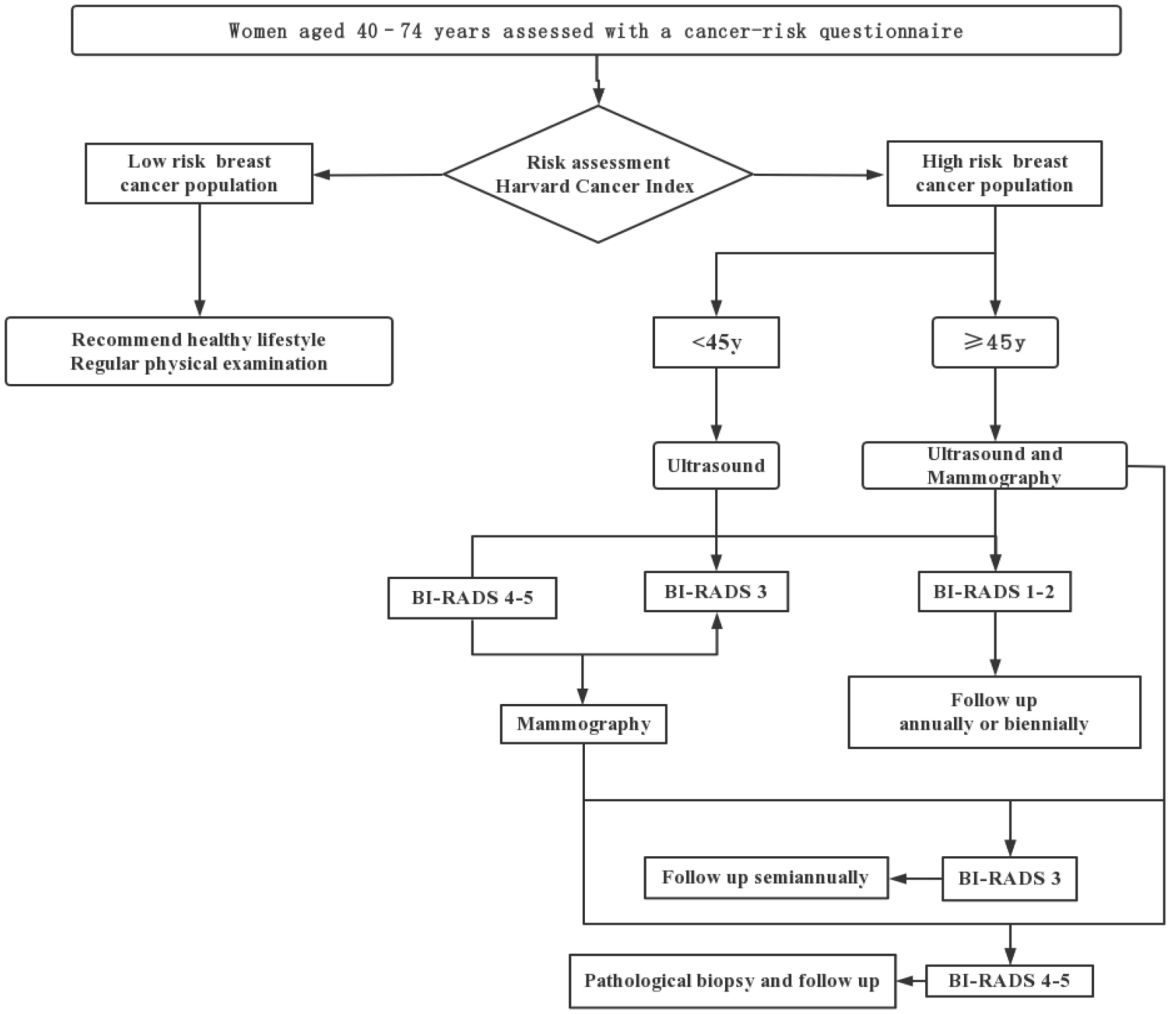
Screening process for breast cancer in Urban Hebei Province.

A total of 84,029 women completed a risk assessment questionnaire during 2016–2020 in Hebei Province. 20,655 were identified as being at high risk of developing breast cancer after the breast cancer risk evaluation. The program screens high-risk women aged 40–44 years by ultrasound and the women with suspected results are further examined by mammography. High-risk women aged 45–74 years screened with both ultrasound and mammography. All suspected results from either method are confirmed with biopsy^18^. Figure 1 present the breast cancer screening process of the high-risk population. Breast cancer individuals in the screening arm can be diagnosed while still asymptomatic and at an earlier stage, but breast cancer is only diagnosed on presentation of symptoms for low-risk individuals.

#### Modeling strategy

We developed a natural history Markov model for breast cancer screening in urban Hebei females using the TreeAge software (TreeAge software Inc. Williamstown, United States of America), to inform a long-term decision model. Our model predicted the lifetime costs and quality-adjusted life years (QALYs) of screening and no screening for urban Hebei females with no previous history of breast cancer, from 40 years to death. We used annual screening frequency in the baseline analysis, and we explored the scenarios of screening biennially and triennially. The screening strategies included ultrasound only, mammography only and combined ultrasound and mammography. Eventually, there were nine screening strategies enrolled in the study. Additionally, the model validation was also performed by comparison the age-specific incidence rate of breast cancer in the “2019 Hebei Tumor Registration Annual Report” and the age-specific breast cancer incidence rate predicted by the model, and the goodness-of-fit test about the two curves was used for fitting.

### Natural history and initial distribution probability

**Figure 2:**
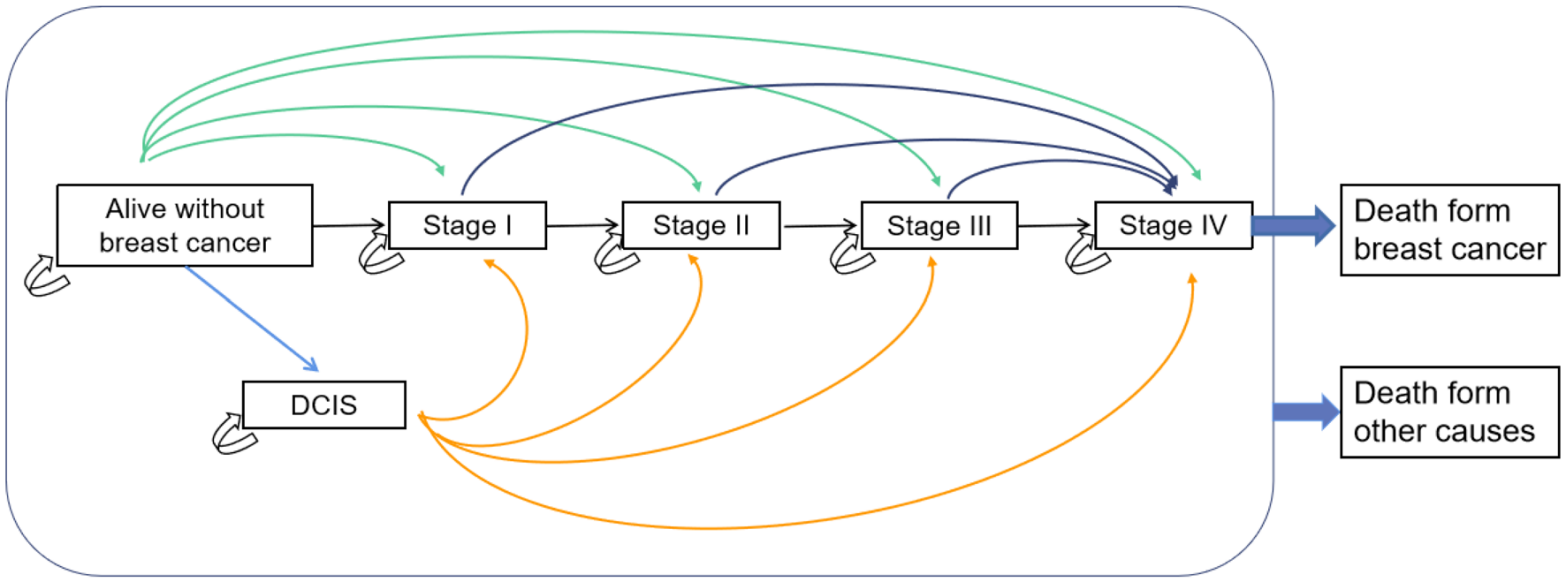
Natural history model for breast cancer progression.

Figure 2 illustrates the various health states and the potential transitions between them. Healthy women can transition to ductal carcinoma in situ (DCIS) or stage I–IV cancer, or remain free of cancer or die. Women with DCIS are at a higher risk of developing invasive breast cancer, or die from causes other than breast cancer or remain the current status. Patients at stage I can progress to stage II, stage III and stage IV in turn. Patients at stage IV can progress die from breast cancer or not, or remain the current status. All women can die from causes other than breast cancer during disease progression, but only patients at stage IV can die from breast cancer. The state progression transition probabilities used in this analysis are from models described in the literature^14^. We estimated the probability of symptoms in non-screened population by calibrating the model as follows. In the non-screening arm, incident cases are only detected on presentation with symptoms; the distribution of incidence cases by stage is therefore a function of the probability of transitions and the probability of symptoms^32,33^. We adjusted the probability of symptoms until the distribution of cases presented at each stage was similar to the distribution of reported incidence cases in Hebei Province (Supplementary Fig. 1). All data were provided in Table 1.

**Table 1:** Parameter values in the Markov model.

Parameter	Baseline	Reference/source
Age-specific incidence of invasive breast cancer (years)		^34^
40–44	68.79 × 10^5^	
45–49	86.23 × 10^5^	
50–54	110.91 × 10^5^	
55–59	90.34 × 10^5^	
60–64	136.96 × 10^5^	
65–69	129.21 × 10^5^	
70–74	100.77 × 10^5^	
75–79	103.81 × 10^5^	
80–84	93.42 × 10^5^	
≥ 85	40.17 × 10^5^	
Age-specific mortality of all-cause; female (years)		^35^
40–44	63.16 × 10^5^	
45–49	112.74 × 10^5^	
50–54	163.58 × 10^5^	
55–59	276.53 × 10^5^	
60–64	449.65 × 10^5^	
65–69	847.15 × 10^5^	
70–74	1537.34 × 10^5^	
75–79	3025.46 × 10^5^	
80–84	5808.32 × 10^5^	
≥ 85	18,516.44 × 10^5^	
Age-specific mortality of female breast cancer; (years)		^35^
40–44	5.52 × 10^5^	
45–49	10.59 × 10^5^	
50–54	12.90 × 10^5^	
55–59	18.72 × 10^5^	
60–64	19.85 × 10^5^	
65–69	22.26 × 10^5^	
70–74	22.08 × 10^5^	
75–79	26.18 × 10^5^	
80–84	38.72 × 10^5^	
≥ 85	70.77 × 10^5^	
Age-specific mortality of non-female breast cancer; (years)		^35^
40–44	57.64 × 10^5^	
45–49	102.15 × 10^5^	
50–54	150.68 × 10^5^	
55–59	257.81 × 10^5^	
60–64	429.8 × 10^5^	
65–69	824.89 × 10^5^	
70–74	1515.26 × 10^5^	
75–79	2999.28 × 10^5^	
80–84	5769.60 × 10^5^	
≥ 85	18,445.67 × 10^5^	
Stage-specific probability of symptoms		Model calibration
Stage I	0.004	
Stage II	0.014	
Stage III	0.038	
Stage IV	0.098	
Average annual progression probability of breast cancer staging		^36^
Stage I–Stage II	0.01	
Stage II–Stage III	0.08	
Stage III–Stage IV	0.21	
RR of invasive cancer from DICS	2.02	^38^
Utility scores		^39,40^
Health status		
Health	1.00	
DCIS	0.76 (0.69–0.83)	
I	0.79 (0.77,0.80)	
II	0.79 (0.78,0.80)	
III	0.77 (0.76–0.79)	
IV	0.69 (0.65–0.72)	
Death	0.00	
Effectiveness of screening		^37^
Ultrasound sensitivity	0.63 (0.53–0.71)	
Ultrasound specificity	0.99 (0.98–0.99)	
Mammography sensitivity	0.86 (0.79–0.93)	
Mammography specificity	0.94 (0.94–0.95)	
Mammography and ultrasound sensitivity	0.95 (0.91–0.99)	
Mammography and ultrasound specificity	0.93 (0.93–0.94)	

### Parameter values in the Markov model

#### Epidemiological and clinical data

We obtained the age-specific invasive breast cancer incidences from the 2019 Hebei Cancer Registry Annual Report^34^. We calculated age-specific mortality from other causes by subtracting age-specific breast cancer mortality rates from the corresponding age-specific all-cause mortality rates^35^. As there is no screening group data in Hebei Province, data reported in literature are used instead^36,37^. All data were provided in Table 1.

#### QALYs

QALYs is a measurement that reflects both length of life and health-related quality of life. It is calculated as the product of the utility score of a particular state of health, defined as a dimensionless number between 1 (perfect health) and 0 (death), and the number of years lived. We identified the utility scores for patients at stage I, II, III and IV from a cross-sectional survey conducted as part of the screening program. The middle value represents the disease state, and the smaller the value, the lower the individual’s quality of life, and the greater the impact of the disease state on the quality of life. As the cancer stage increases, the health effect value decreases.

#### Effectiveness of screening

We used the sensitivity (probability of positive diagnosis if diseased) and specificity (probability of negative diagnosis if not diseased) values from a multi-center Breast Cancer Optimized Screening Program in China, screening strategies were compared, which are different combinations of mammography and ultrasound.

#### Costs

This study adopts the discount rate of 3% used in cost-effectiveness analysis as majority literature studies reported^18^, and the monetary unit of all costs in this paper is expressed in the form of ¥RMB(Yuan). Data describing the costs of management costs, screening (whether ultrasound or mammography, or ultrasound plus mammography) and biopsy were available from the screening program. We also obtained the screening costs and treatment costs by stage in the study. The screening cost refers from CanSPUC program, including ultrasound and mammography screening technology costs and management costs. The treatment cost data comes from the CNBCSP-Urban, including medical expenses and non-medical expenses. Medical expenses refer to expenses including bed fees, examination fees, examination fees, treatment fees, surgical fees, laboratory examination fees, nursing fees, drug fees, and so on. The non-medical expenses refer to the patients’ accommodation, transportation, and extra meals. Economic resources consumed by non-health care sectors. All cost data has been discounted to 2019 (Table 2).

**Table 2:** Related cost parameters of Markov model in breast cancer screening.

Cost	Variables	Baseline (Yuan, CN)	Source
Screening costs	Ultrasound	76	CNBCSP-Urban*
	Mammography	219	
	Mammography and Ultrasound	295	
	Management costs	22	
Treatment costs	DCIS	14,900	^18,41,42^
	I	61,600	
	II	67,725	
	III	78,733	
	IV	108,710	

CNBCSP-Urban*—Data source form the breast cancer screening program in Urban Hebei.

### Analysis

Average cost-effectiveness ratios (ACERs) were estimated as the ratios of the additional costs of the screening strategies to the QALY compared to no screening. Incremental cost-effectiveness ratios (ICERs) were calculated based on the comparison of a lower cost strategies to the next more expensive and effective strategies after excluding dominated strategies and extendedly dominated strategies^43^. The willingness-to-pay (WTP) threshold was estimated to be three times the gross domestic product (GDP) per capita in China in 2019 (¥213,000Yuan). An ICER of less than ¥ 213,000Yuan/QALY is therefore an indication that the breast cancer screening for urban Hebei women aged 40–74 years, compared to no screening, is cost-effective.

In this study, sensitivity analysis was used to explore the factors that influence the screening program for breast cancer. When other parameters remain unchanged, by changing the value of a certain influencing factor within a predetermined range, the influence degree of the factor was used to examine the stability of the model. The factors included in the sensitivity analysis in this study included discount rate, health utility value, sensitivity, specificity and treatment costs. The first four factors were enrolled with 95% CI, and the treatment cost uses 20% as the possible range of variation. Tornado diagram was used to demonstrate the influencing factors of ICER in breast cancer screening program.

### Ethics approval and consent to participate

The use of the data from the breast was approved by the Ethics Informed Committee of the Fourth Hospital of Hebei Medical University (Shijiazhuang, Hebei, China), and all methods were performed in accordance with the approved guidelines. Written informed consent was obtained from all subjects or from their next of kin if the patients were deceased.

## Results

### Baseline of breast cancer screening in Hebei, 2016–2020

In the screening period, there were 84,029 female individual participated the cancer screening program in Urban Hebei, 20,655 were estimated as high risk of breast cancer with the high-risk rate of 24.58%. The highest rates of breast cancer were 40–64 age groups, with all the high-risk rates above 20%. Among the high-risk women, 13,392 individuals undertook the breast cancer screening with the total compliance rate of 64.84%. The relative high compliance rates were 45 to 64 years old with the rates of 64.27%, 66.49%, 71.53% and 67.96%, respectively, as shown in Table 3.

**Table 3:** Baseline breast cancer screening in Urban Hebei Province.

Age groups	Participants		Participants of high risk for Breast cancer			Participants undertaking screening		
	N	Pro (%)	N	HRR (%)	Pro (%)	N	CR (%)	Pro (%)
40–44	10,339	12.30	2674	25.86	12.95	1426	53.33	10.65
45–49	13,231	15.75	4257	32.17	20.61	2736	64.27	20.43
50–54	14,462	17.21	4324	29.90	20.93	2875	66.49	21.47
55–59	13,024	15.50	3527	27.08	17.08	2523	71.53	18.84
60–64	14,702	17.50	3199	21.76	15.49	2174	67.96	16.23
65–69	11,963	14.24	2017	16.86	9.77	1242	61.58	9.27
70–74	6308	7.50	657	10.42	3.17	416	63.32	3.11
Total	84,029	100.00	20,655	24.58	100.00	13,392	64.84	100.00

*N* number of cases, *Pro* proportion, *HRR* high risk rate, *CR* compliance rate.

### Detection rates of risk-based breast cancer screening

**Figure 3:**
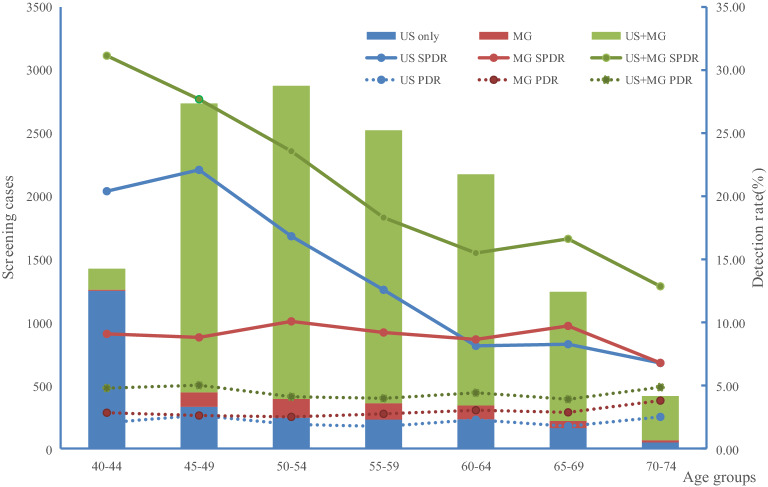
Detection rates for breast cancer by ultrasound, mammography and combined screening: *US* ultrasound, *MG* mammography, *SPDR* suspicious positive detection rates, *PDR* positive detection rates.

Among high-risk breast cancer population undertook ultrasound, mammography and combined screening, the suspicious positive detection rates (SPDR) (BI-RADS-3) were 15.00%, 9.20% and 19.30%, respectively. The SPDR of mammography screening generally demonstrated a steady trend with age. The highest SPDR with 22.09% presented in 45–49 age group for the ultrasound screening. The SPDR detection rates demonstrate a decreased trend for the combined ultrasound and combined screening method. The positive detection rates (PDR) (BI-RADS-4 and 5) of women who screened by ultrasound, mammography and combined screening were 2.11%, 2.76% and 3.83%, respectively. For ultrasound screening, the highest detection rate was 2.63% in the 45–49 age group. Inversely, the highest detection rates were 3.80% and 4.86% both in the 70–74 age group for mammography and combined screening, respectively (Fig. 3).

### Comparison of the effects between screening and no screening

**Figure 4:**
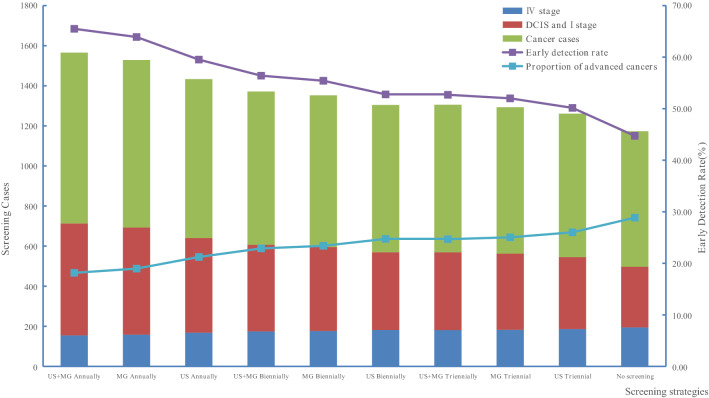
The morbidity and mortality for breast cancer at the end of the 45 years. *US* + *MG* combined ultrasound and mammography screening, *MG* mammography screening, *US* ultrasound screening.

As is shown in Fig. 4 and Table 4, with the same screening method, the highest detection rate of early diagnosis and number of breast cancer detected, accompanied by the lowest proportion of advanced cancers and the least number of deaths were performed by the annual screening interval. Annual screening by ultrasound, mammography and combined, the early diagnosis rates were 55.53%, 60.68% and 62.47%, the proportion of advanced cancer were 17.20%, 15.85% and 15.36%, respectively. Compared with any screening method, the no screening population had the lowest rate of early diagnosis rate and the least number of breast cancer detected, the proportion of advanced cancers (25.15%) and the number of deaths was the highest inversely.

**Table 4:** The comparison of the effect among screening and no screening strategies.

Variables	Frequency									
	No screening	Ultrasound			Mammography			Combined		
		Annual	Biennial	Triennial	Annual	Biennial	Triennial	Annual	Biennial	Triennial
Summary of morbidity and mortality at the end of the 45 years in each screening program										
DCIS + I stage	282	452	367	331	517	400	352	541	413	360
IV stage	163	140	149	149	135	147	147	133	146	146
Cancer cases	648	814	766	747	852	785	761	866	791	765
Total deaths	71,776	71,609	71,659	71,676	71,573	71,640	71,663	71,558	71,632	71,658
Early diagnosis rate (%)	43.52	55.53	47.91	44.31	60.68	50.96	46.25	62.47	52.21	47.06
Proportion of advanced cancers (%)	25.15	17.20	19.45	19.95	15.85	18.73	19.32	15.36	18.46	19.08
Morbidity and mortality of each screening group (person year)										
DCIS	8213	15,710	10,739	8846	19,483	12 677	10 085	20,947	13,428	10,565
I stage	13,808	17,650	17,215	17,037	17,985	17 394	17 148	18,119	17,462	17,193
II stage	7829	9268	11,399	12,206	7653	10 568	11 673	7024	10,248	11,471
III stage	7669	6519	7487	7852	5779	7111	7609	5496	6964	7518
IV stage	11,793	8891	9549	9794	8395	9288	9630	8203	9193	9567
No cancer deaths	1,666,219	1,675,473	1,674,074	1,673,479	1,676,580	1,674,663	1,673,844	1,677,008	1,674,889	1,673,989
Cancer deaths	61,270	43,597	46,648	47,898	41,230	45,402	47,112	40,311	44,922	46,813
Total deaths	1,727,489	1,719,070	1,720,722	1,721,377	1,717,810	1,720,065	1,720,956	1,717,319	1,719,811	1,720,802
Development of each screening group in the next 45 years										
Early-stage cases	22,021	33,360	27,954	25,883	37,468	30,071	27,233	39,066	30,890	27,758
Early diagnosis rate increased (%)	–	51.49	26.94	17.54	70.15	36.56	23.67	77.40	40.28	26.05
IV stage cases	11,793	8891	9549	9794	8395	9288	9630	8203	9193	9567
IV stage incidence decreased (%)	–	24.61	19.03	16.95	28.81	21.24	18.34	30.44	22.05	18.88
Breast cancer deaths	61,270	43,597	46,648	47,898	41,230	45,402	47,112	40,311	44,922	46,813
Breast cancer mortality decreased (%)	–	28.84	23.86	21.82	32.71	25.90	23.11	34.21	26.68	23.60

With the increasing in the person year of DCIS and stage I breast cancer, the person year incidence of stage IV breast cancer cases and the death rate of breast cancer were both decreased. Whatever any screening methods, the person year number of patients with stage IV incidence and the number of deaths from breast cancer were lower than those of the no screened group (11,793-person year and 61,270-person year).

The different screening methods at the same screening interval yield varied. The combined screening performed the highest decreased rates for IV stage incidence and breast cancer mortality with 30.44% and 34.21%, respectively, followed by the mammography screening with the rates of 28.81% and 32.71%, the ultrasound screening showed the lowest rates of 24.61% and 28.8%. Whatever biennial or triennial screening, the rates were demonstrated the same trends as annual screening.

The different screening interval at the same screening method yield differently as well, the best effectiveness was annual screening, followed by biennial screening, the triennial was the worst. Although compared with the no screening group, the improvement of various indicators in the biennial and triennial screening interval is not as great as that of the annual screening interval, the early diagnosis rate has also increased, and both the incidence rate of stage IV breast cancer and the mortality rate of breast cancer have been reduced by more than 15.00% and 20.0% respectively.

### Cost-effectiveness analysis

Table 5 reports the discounted lifetime costs, QALYs, ICERs and ACERs. Overall, compared with no screening, the other nine risk-based breast cancer screening strategies yielded higher QALYs, cost more expensive simultaneously. Comparing with no screening strategy and in the exploration of various scenarios under the WTP threshold, annual, biennial or triennial ultrasound screening strategies and annual mammography screening strategy were regarded as the cost-effectiveness strategies with the ACERs of ¥116,176.15/QALY, ¥148,463.27/QALY, ¥170,038.67/QALY and ¥188,963.87/QALY.

**Table 5:** The cost-effectiveness ratios of high risk-based breast cancer screening strategies in Urban Hebei Province.

Strategy	ACER					ICER		
	Cost	Benefit	Δ Cost*	Δ Benefit*	ACER	Δ Cost	Δ Benefit (QALY)	ICER (per QALY)
No screening	26,611.76	17.71	–	–	–	NA	NA	NA
US screening triennially	31,138.89	17.73	4527.13	0.03	170,038.67	ED	ED	ED
US screening biennially	31,174.57	17.74	4562.81	0.03	148,463.27	ED	ED	ED
US screening annually	31,366.35	17.75	4754.59	0.04	116,176.15	0.04	4754.59	116,176.15
MM screening triennially	34,369.99	17.73	7758.23	0.03	265,554.88	D	D	D
MM screening biennially	34,710.45	17.74	8098.69	0.03	232,435.04	D	D	D
MM screening annually	35,833.23	17.75	9221.47	0.05	188,963.87	0.01	4466.88	567,261.63
US + MM screening triennially	36,045.47	17.74	9433.71	0.03	312,164.66	D	D	D
US + MM screening biennially	36,620.15	17.74	10,008.39	0.04	274,677.25	D	D	D
US + MM screening annually	38,266.52	17.76	11,654.76	0.05	224,757.08	0	2433.29	796,560.57

Incremental is denoted as ‘Δ’. Benefit and incremental benefit are measured in quality-adjusted life-years (QALYs). The incremental cost-effectiveness ratio (ICER) is measured in cost per QALY. Incremental values are not reported for dominated (‘D’) or extendedly dominated (‘ED’) strategies; Average cost-effectiveness ratios (ACERs) were estimated as the ratios of the additional costs of the screening strategies to the QALY compared to no screening.

‘*’Means the comparison with the no screening strategy.

According to the WTP threshold, out of nine breast cancer screening strategies, there are three alternative undominant strategies, including annual ultrasound screening, annual mammography screening and annual combined ultrasound and mammography screening. Based on the cost effectiveness evaluation standard and the largest effect principle, annual ultrasound screening strategy was the most cost-effectiveness and yield the largest effect with obtaining the benefit of 17.75QALYs and ICER of ¥116,176.15/QALY. Although annual mammography screening and annual combined ultrasound and mammography screening strategies were undominant strategies, the ICERs were more than the WTP threshold with 567,261.63/QALY and 796,560.57/QALY.

### Sensitivity analysis

**Figure 5:**
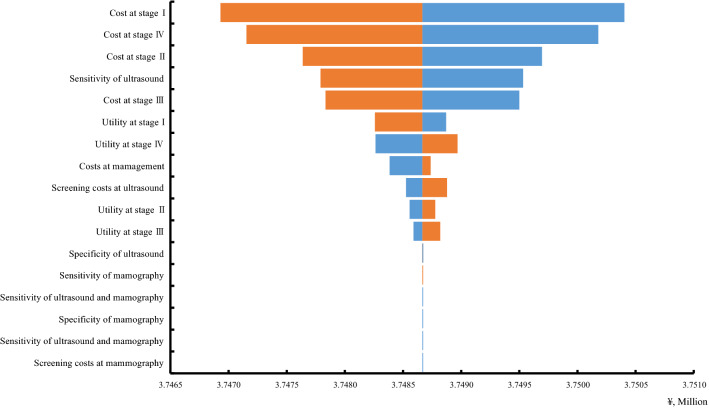
Tornado diagram.

In sensitivity analysis, which is illustrated in a tornado chart as in Fig. 5, the cost of stage I was identified as the most important driver of cost-effectiveness for breast cancer screening programs, followed by the cost of stage IV, cost of stage II and sensitivity of ultrasound. The sensitivity and specificity of ultrasound were also key determinants of the ICER. Interestingly, the sensitivity and specificity of mammography and combined did not show a significant impact on cost-effectiveness in the chosen model.

## Discussion

In the several decades, Hebei Province suffered from the relative heavy breast cancer burden in urban, which was higher than the average level in urban China^34^. By the screening strategy combined ultrasound and mammography could gain a higher chance of detecting suspicious positives and positive cases for breast cancer. However, when exploring breast cancer screening programs, the input–output ratio must be taken into account, which is significant to evaluate related health economic effect for screening^44^. Up to know, to our knowledge, there were few studies had reported the breast cancer screening and detection baseline in Hebei, and the current study was even the first time to research the health economics evaluation of breast cancer screening program in Urban Hebei Province.

In Hebei Province, findings from this study could help to identify that the high-risk rate of breast cancer was 24.58% in 2016–2020, which was consistent with the previous report in Hebei with the high-risk rates of 27.31% in 2018–2019^45^, which were relatively higher than the latest data about the preliminary of cancer screening program in Urban China with the rates of 19.47% in 2012–2016 and 21.54% in 2013–2017, respectively^46,47^. Comparing with other urban regions in China, high-risk rate also higher than Zhejiang (12.56%, 2013–2018) and Hunan Province (19.45%, 2012–2018)^48,49^. It deserves to be noted that the overall positive rate of breast cancer screening was high in a high-risk population in urban China, with around 44% participants having benign or potential malignancies^46^. In our study, the detection rate for suspicious malignancy by ultrasound was 2.11%, the results were in line with previous researches conducted in Hebei with 2.46%^45^, which were both higher than Cancer Screening Program in Urban China baseline detection rate in the period of 2012–2016 and other previous researches conducted in China^20,46,50^. However, the current study demonstrated that the detection rates of positive lesions by mammography only is superior to ultrasound only for breast cancer screening for high-risk women in Hebei Province, which was consistent with Beijing city and a Chinese cohort study^51–53^, but contrary with some previous researches conducted in China. Differences in detection rates for regions may be due to various in local economy, environment and demographic structure, or differences in reporting time^20,54^.

In majority breast cancer screening programs, mammography was regarded as the main screening strategies. However, the diagnostic accuracy of mammography for breast cancer was not equal in all women. The overall sensitivity of mammography for detecting breast cancer was around 85%, but it dropped dramatically to 47.8–64.4% for women with dense breast tissue^46^. There have been several economic evaluations of mammography to screen for breast cancer as the main strategy in Chinese females. One of researches by Wong reported the least costly, nondominated screening option was screening from ages 40 years to 69 years^36^. Woo et al. reported that the ICER was 90 771USD/DALY when screening biennially for the age group of 50–74 years old^55^. Wu et al. reported that it is less cost-effective to use mammography screening alone in Shanghai, China^56^. Our study demonstrated that, comparing with no screening, the cost-effectiveness program was mammography screening annually, which was consistent with previous study^36^. Comparing with other countries’ researches, annual screening by mammography generated an ICER of $565,912/QALY in Canada, which is considerable uncertainty about the incremental cost-effectiveness of a WTP threshold^57^, In Germany, MR-mammography resulted with an ICER of $45,373.94/QALY, which was higher than the WTP as well^58^. An American study by Shis et al. reported that baseline mammography screening yielding an ICER of $36,200/QALY between 50 and 75 years old, which still higher than the WTP^59^. Conversely, our current study result showed that annual screening by ultrasound is the most cost-effective with an ICER of ¥116,176.15/QALY, which was consistent with several Chinese researches, such as Sun et al., which reported screening by ultrasound could be regarded as the primary method for breast cancer screening in Chinese females, while screening by mammography could only be used in some eastern economically developed regions^60^. Another Chinese study reported that, comparing with never screening, biennial screening with clinical breast examination and breast ultrasound was the most cost-effective breast cancer screening strategy, with the cost of saving related QALY would be ¥91,944^37^. Additionally, the Beijing Cancer Screening Prospective Cohort Study reported ultrasound alone (48,323 RMB ($7550)) was the most cost-effective methods for breast cancer screening than other screening strategies^61^.

A Markov model was used to explore and assess the effective and cost-effectiveness of breast cancer screening in Hebei Province, to get proper estimates; all input parameters were obtained based on systematic literature searches. To our knowledge, there have few studies combined mammography and ultrasound screening reported so far in China. Our results from the cost-effectiveness analysis suggest that the risk-based breast cancer screening program is cost effective over the no screening group in Hebei, which was constant with previous studies^18,62^. Our baseline model of annual screening by ultrasound only yielded an ICER of ¥116,176.15/QALY, lower than the WTP threshold of ¥213,000/QALY, which was considered as the most cost-effective screening strategy. Therefore, may imply that ultrasound screening has been proposed as a possible, more favorable alternative strategy for high risk women in Hebei Province^63^. Sensitivity analysis showed that although cost is the most effect factor in our study, within the value range of the variables in this study, whatever how changes in the other variables, there was no fundamental impact on ICER, and ICER was still below the willingness to pay threshold, which was consistent with a Germany study^64^.

### Limitations

The current study is based on the breast cancer screening program and established a Markov model that simulates the female population in urban Hebei. This model uses as many parameters as possible in this study to better fit the epidemiological characteristics and cost of breast cancer in Hebei women. At the same time, the study proposed that annual ultrasound screening for positive patients is the optimal strategy, which provides a basis for decision-making for the selection of breast cancer screening programs for women in urban Hebei, as well as provides breast cancer screening strategies in other regions where economic level and incidence of breast cancer are similar to Hebei province. However, a few limitations of this study need to be noted. Firstly, this study used model-based estimates based on assumptions. The model assumes 100% attendance and compliance with breast cancer screening and follow-ups, which is not representative of the real-world situations. Breast cancer screening among urban population in China from 2013 to 2018 and Hebei province from 2018 to 2019 showed that 55.3% and 64.4% of women aged 40–74 years old have attended screening^45,65^. Secondly, the study explored the impact of access to treatment on the overall results, suggesting that if not all detected cases go on to receive treatment, the screening is less cost-effective. Chinese patients need to pay on nearly 36% of total medical expenses, which could limit access to medical treatment for some women who have been diagnosed with breast cancer. Some women may also decide not to seek medical treatment if they are asymptomatic, such delay in the treatment of cancer can have adverse consequences on outcome and finally reduce the cost-effectiveness of a screening^66^. Thirdly, our study only contained the cost-effectiveness of various combinations of screening methods and interval in urban regions, due to the age-specific incidence was lower in rural regions than in urban regions, we were unable to distinguish effects across rural regions in Hebei Province yet. Future research is required to investigate differences between urban–rural regions^67^. Lastly, it should be noted that any Markov decision model should be validated using external empirical data. However, the screening program still requires long-term follow up to provide this empirical data. Moving forward, continuous follow up of the target population is needed over an extended period of time to facilitate a long-term evaluation of its effectiveness.

## Conclusion

In general, although it was not cost effective, ultrasound combined mammography screening strategy had a higher chance of detecting suspicious positives and positive cases. High-risk population-based breast cancer screening by ultrasound annually is the most cost-effective in Urban Hebei Province. However, considering the large geographical and socioeconomic disparities across, tailored screening strategies are required to further improve the effectiveness of breast cancer screening among Hebei women.

## Supplementary Information

Supplementary Figure 1.

## Data Availability

The datasets generated and analyzed during the current study are not publicly available but are available from the corresponding authors.

## Abbreviations

QALY: Quality-adjusted life year
ICER: Incremental cost-effectiveness ratios
CanSPUC: Cancer Screening Program in Urban China
CanSPRC: Cancer Screening Program in Rural China
DCIS: Ductal carcinoma in situ
ACER: Average cost-effectiveness ratio
