# Supplementary material for: Cost-effectiveness evaluation of risk-based breast cancer screening in Urban Hebei Province
Source: Sci Rep. 2023 Feb 27;13:3370. doi: 10.1038/s41598-023-29985-z (PMC9971026; doi:10.1038/s41598-023-29985-z)
Supplement: Supplementary file 1 — Supplementary Figure 1. [file 41598_2023_29985_MOESM1_ESM.docx]

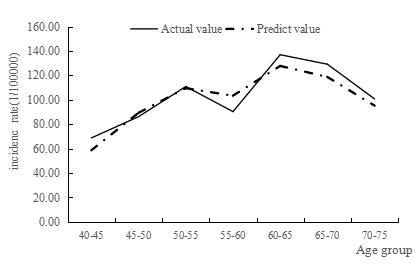


Supplementary figure 1 Comparison of predicted age-specific incidence rates and actual age-specific incidence rates curve of female breast cancer in Hebei Province.
